# Music Lessons for the Study of Affect

**DOI:** 10.3389/fpsyg.2021.760167

**Published:** 2021-11-29

**Authors:** Robert R. McCrae

**Affiliations:** Retired, Gloucester, MA, United States

**Keywords:** affect dynamics, vitality affects, volitional affects, affect transitions, emotion threads

## Abstract

Some accounts of the evolution of music suggest that it emerged from emotionally expressive vocalizations and serves as a necessary counterweight to the cognitive elaboration of language. Thus, emotional expression appears to be intrinsic to the creation and perception of music, and music ought to serve as a model for affect itself. Because music exists as patterns of changes in sound over time, affect should also be seen in patterns of changing feelings. Psychologists have given relatively little attention to these patterns. Results from statistical approaches to the analysis of affect dynamics have so far been modest. Two of the most significant treatments of temporal patterns in affect—sentics and vitality affects have remained outside mainstream emotion research. Analysis of musical structure suggests three phenomena relevant to the temporal form of emotion: affect contours, volitional affects, and affect transitions. I discuss some implications for research on affect and for exploring the evolutionary origins of music and emotions.

## Introduction

How is it that rhythm and melodies, although only sound, resemble states of the soul? –Aristotle, *Problemata*, c. 19, cited in Schopenhauer, 1969, (p. 260).

How might psychological research be steered in productive directions by examining the accumulated experience of composers and music theorists? –Rozin and Rozin, 2018, (p. 566).

Infants respond to musical cues, and some form of music is found in every culture; the tendency to create, perceive, and appreciate music must be part of evolved human nature. But since Darwin (1871), the reasons for this have remained a mystery (McDermott, 2008). Music may be a mere byproduct of other evolved adaptations (Pinker, 1997), or a “transformative technology” (Patel, 2010, p. 401) that, once invented, became an essential feature of human society. Music may have evolved through sexual selection (as the custom of serenading suggests); it may confer adaptive advantages by promoting bonding with infants (through lullabies) or social cohesion (Savage et al., 2020) among groups (as through national anthems).

But none of those evolutionary hypotheses explains the unique connection of music to emotion. At least two other perspectives, however, do. The first is the idea that music evolved from vocalizations that were used to communicate emotions (Snowden et al., 2015), perhaps as part of a pre-linguistic precursor of both music and language (Darwin, 1871; Masataka, 2009). Just as names presumably evolved from vocative addresses to individuals into nouns that could refer to the individuals in their absence, so sounds that communicated a current emotional state (fear, distress, love) came to express (or symbolize, as Langer, 1957, would argue) these affects themselves.

The second is a proposal by Perlovsky (2010) that music co-evolved with language to compensate for the hypertrophy of cognition that language facilitated. Perlovsky, who had an intriguing if idiosyncratic theory of psychology and culture, argued that language promotes a focus on conceptual thinking, leaving behind the instinctual, emotional, and behavioral aspects of the person. Without a synthesis of all these aspects, people lack a sense of meaning in life. Music and musical emotion evolved because it restores the unity of the self and thus the will to live.

These arguments provide an evolutionary rationale for the widespread belief that music is the language of emotion. The emotionality of music is not a cultural convention, invented in the Romantic era of Western music; it is the *raison d’être* of human music. If so, then music ought to be as central a concern to research on emotion as language is to research on cognition. It has not been. In this essay I review what is known about the expression of affect in music and point to new directions for emotion research suggested by the psychology of music. In particular, I focus on the temporal unfolding of affect that parallels the dynamic flow of music. I will also touch briefly on some implications for understanding the evolution of music and of emotions.

### The Psychologies of Music and Emotion

There is an extensive literature on music and emotions (Juslin and Sloboda, 2001; Swaminathan and Schellenberg, 2015; Juslin, 2019), and contemporary researchers in this subfield rely heavily on mainstream models of affect and emotion. However, there appears to be little reciprocal influence of music research on general theories of emotion, which is perhaps unfortunate. Hevner (1936) reported a circular arrangement of emotion terms to be used in rating musical samples; its vertical axis was marked by *merry* versus *mournful*, and its horizontal axis contrasted *vigorous* with *serene*. This scheme appears to have been ignored until, half a century later, Russell (1980) offered his circumplex model of valance and arousal.

As Hunter and Schellenberg (2010) pointed out, much research on music and emotions has quantified the latter using Russell’s (1980) two dimensions. Although it is undeniable that valence and arousal are important characteristics of affect, there is a certain irony in the reduction of the powerful, subtle, and ever-changing expression of affect in music to two-dimensional ratings—it is as if one tried to analyze Van Gogh’s *Starry Night* with a color wheel. Somewhat more elaborate models based on sets of discrete emotions have also been used (Eerola and Vuoskoski, 2011, 2013), but a simple categorical judgment of the emotion portrayed in a passage of music cannot capture the subtleties of the flow of music. Surely a more dynamic theory of emotions is needed, and a theory that can adequately describe the feelings portrayed in music should be of value in understanding all emotional experience. If, as Pratt (1952) argued, “music sounds the way emotions feel” (p. 26), then emotions must feel the way music sounds, and musical analysis must offer insights into affective experience.

Although the psychology of music has had a limited influence on general theories of emotion, music itself has played a major role in research on affective neuroscience. In these studies, neuroimaging and electrophysiological techniques are used to identify areas of the brain that are activated by emotions. Music has frequently been used to induce these emotions (e.g., Blood and Zatorre, 2001; for reviews, see Habibi and Damasio, 2014; Koelsch, 2014), and effects have been documented that depend in part on features of the music (e.g., consonant vs. dissonant, happy vs. sad) and in part on characteristics of the listener (e.g., musical training, music preferences). Koelsch (2013) has pointed to several advantages of using music as a stimulus in this research, including the fact that it can be used to study the time course of affective processes over short (seconds) and longer (minutes) intervals.

There is controversy, however, over whether the feelings induced by music are genuine emotions—and thus whether they are informative about how the brain processes emotion in daily life. Konečni, 2008, (p. 115) argued that, at best, “music may induce low-grade basic emotions through mediators” such as personal associations (e.g., of a song with the loss of a loved one). Koelsch (2014) countered by citing evidence that music affects endocrine responses, facial expression, and action tendencies as well as subjective feeling, and thus “music can evoke real emotions” (p. 178).

However, the affective experience derived from music surely differs somehow from that of everyday life, which is thought to be generated by appraisals of the valence and personal relevance of events (Ellsworth and Scherer, 2003). The distinction between what is felt when listening to music and what is felt in real life is underlined by the curious fact that people often enjoy sad, even tragic music (Sachs et al., 2015); people do not enjoy tragedy when it befalls them. The only class of emotions that music clearly generates are the esthetic emotions such as awe and esthetic chills (McCrae, 2007; Konečni, 2008; Sherer and Zentner, 2008). Here the music is appraised as being beautiful or moving, and the listener shows a distinctive emotional response. Reybrouck and Eerola (2017) pointed out that the esthetic experience has perceptual, cognitive, and affective aspects, and that esthetic emotions are “more contemplative, reflected and nuanced” (p. 9) than core affects and basic emotions. One may be in awe of a Bach fugue, but only if one has acquired knowledge of fugal techniques and become intimately familiar with the music.

## Preliminary Issues

There are several different ways in which music and emotion are related (Juslin and Sloboda, 2001). As noted, music may induce basic emotions in listeners (Scherer, 2004; Zentner et al., 2008): A lively jig may make one merry; a dirge can make one sad. Music yields the pleasures of esthetic emotions, as well as the far subtler satisfactions and surprises that attend the fulfillment or disappointment of musical expectations (Meyer, 1956, 2001). Music may have therapeutic properties, particularly in the treatment of depression (Harris, 2019), and music preferences are related to the personality dimensions that help shape emotions (Greenberg et al., 2016).

However, I am concerned here with none of those issues. Instead, I will consider how music *expresses* emotion, as languages express ideas. Zentner et al. (2000) pointed to the importance of the distinction between what emotions listeners *felt* and what they *perceived* in response to music. It is the latter that is of interest here, because what listeners perceive is what music expresses. This article views music not as a cause of emotions but as an instructive model of human affect.

As a formal philosophical conception, the idea that music expresses emotion can be traced to Schopenhauer (1969), who distinguished music as the direct expression of the will, “the most secret living, longing, suffering, and enjoying, the ebb and flow of the human heart” (p. 321). This view was elaborated by Langer (1957), who argued that “[b]ecause the forms of human feeling are much more congruent with musical forms than with the forms of language, music can reveal the nature of feelings with a detail and truth that language cannot approach” (p. 235). Recently this position has been championed by Zbikowski (2010).

In what sense can one say that music “expresses” emotion? In much music (songs, film scores, tone poems), it might be argued that music illustrates the emotions felt by the singer or the characters; the music is about the passion of Romeo and Juliet or the grandeur of the Moldau.

But absolute music is self-referential, being only about itself, and yet listeners—even those who resist the temptation to fabricate a storyline for the music—can perceive its emotional content. Zangwill (2004), who believed that music “should not be understood in terms of emotion” (p. 29), nevertheless admitted that it “possesses important qualities that we *describe* in metaphorical emotional terms” (p. 24). This article identifies some of those qualities and proposes that they have parallels in human affect.

I have used the broad term *affect* in the title because some psychologists (e.g., Konečni, 2008)—unlike laypersons— make a sharp distinction between mood and emotion, seeing the former as a relatively long-lasting affective state (e.g., free-floating anxiety), and the latter as a brief and intense response to an event directly relevant to one’s goals and values (e.g., anger in response to an insult). I will use *affect*, *emotion*, and *feeling* interchangeably to refer to affective experience, and will include a broad range of affects that goes beyond the small list of basic emotions (Izard, 2009) to include such feelings as effort, adoration, interest, dominance, and abruptness (Cowen et al., 2019). But I will not deal with the cognitive, physiological, or relational aspects of emotions. Thus, my concern is with what Izard (2009) called, oddly but aptly, “emotion feelings.” (Damasio and Carvalho, 2013, also use the term *feelings* to refer to the mental representation of emotion, but they include under that term consciousness of drives such as hunger and pain, with which I will not deal here).

In this article I will refer chiefly to Western music of the past few centuries, which has been the focus of most research. However, if the link between music and emotion is evolved, it should appear across cultures. Although the literature is scant, it seems to support this position. For example, Balkwill and Thompson (1999) showed that Western participants unfamiliar with Indian music were able to identify joy, sadness, and anger as expressed in ragas.

Finally, it must be recalled that music has many attributes other than emotional expression, and that listeners may focus on these instead. Music can be a purely sensuous experience, a calming background, a template for dancing, or the object of intellectual analysis and interpretation. These and other functions of music will not be considered here.

## Affective Tone

Perhaps the most extensively researched topic on musical expression concerns the overall affective tone of a piece of music (e.g., Gerardi and Gerken, 1995; Balkwill and Thompson, 1999; Eerola and Vuoskoski, 2011). Pratt (1952) demonstrated that laypersons can correctly identify the mood of a passage of music: Given the choices of *stately, spritely, wistful*, or *vigorous*, 91% labeled the opening of Brahms’s First Symphony *stately*, and 99% called Mendelsohn’s “A Midsummer Night’s Dream” Overture *sprightly*. Many researchers (e.g., Thayer, 1986; see Table 10.2 in Gabrielsson and Lindström, 2001, for a review) have attempted to identify the particular musical features—tempo, articulation, mode, volume, and so on—that express different emotions. Results are consistent across studies and would be unsurprising to any musician. Using computer analyses of sound features in film scores to predict emotion ratings by non-musician judges, Eerola (2010) found that happiness was associated with high pitch, rapid tempo, bright timbres, and major mode, whereas sadness was expressed by “rich timbre, slowly changing key centers, and a stable, unchanging register” (p. 220). Sloboda and Juslin (2001) described such musical qualities as *iconic* sources of emotion, and Snowden et al. (2015) showed parallels across species: “the acoustic structures involved must have similar effects on the nervous system of both human infant and animal recipients” (p. 24).

Pratt (1952) and Langer (1957) argued that these associations reflect certain formal similarities between music and affect—the phenomenon remarked on by Aristotle in the *Problemata.* Zbikowski (2010) argued that it is the fundamental cognitive capacity for analogy that underlies the ability to perceive music in emotional terms. Attractive as this argument is, it is incomplete, because it does not explain the basis of the analogy. There is a related issue long studied by philosophers and psychologists: cross-modal or amodal perception. Even infants intuitively grasp that what is seen and what is felt is the same object, despite the difference between vision and touch (Stern, 1985). To paraphrase Pratt (1952), we might say that a ball looks like a ball feels. This is possible because there is some set of essential characteristics of objects (perhaps size, orientation, motion) that can be perceived interchangeably through different senses. It is not entirely clear what essential characteristics are shared by sounds and feelings.

In some cases the basis for the analogy is understandable. Depressed patients may show psychomotor retardation, and funeral marches are invariably slow. The conflict that characterizes the experience of anger parallels the clash of dissonant harmonies. Certainly the degree of arousal in an emotion can be linked to the energy of the music—its tempo and volume; and the aggressive impulses generated by anger have a formal parallel in accented, staccato notes. But by what analogy is a major chord perceived as happy and a diminished seventh chord as spooky? Why does a *bright* timbre (itself so-called in analogy to light) resemble a cheerful mood? Answering such questions may deepen our conceptualization of affect as well as music.

## Studies of Affect in Time

As Reybrouck and Eerola, 2017, (p. 5) noted, music is a temporal phenomenon, “with the experience of time as a critical factor.” The same might be said for the affective aspect of emotion. Watson (2000) argued that “waking consciousness is experienced as a continuous *stream of affect*, such that people are always experiencing some type of mood” (p. 13). Barrett et al. (2007) concurred, but noted that emotion may be back- or foregrounded in consciousness by attention. Obvious as these observations may be, until recently they had not inspired much research.

### Temporal Studies of Affect

Some exceptions should be noted. Watson (2000) has reported an extensive program of research on mood, including daily, weekly, and seasonal variation. In a measure of emotional intelligence, Mayer et al. (2000) included subscales that assessed the ability to predict sequences of emotional events: progressions (as from anger to rage) and transitions (as from fear to tranquility through acceptance). Appraisal theories (e.g., Lazarus, 1966; Ellsworth and Scherer, 2003; Moors et al., 2013) see emotion episodes as unfolding processes, with appraisal of an event and of our ability to deal with it leading to an emotional state that affects attention, memory, and physiological arousal in preparation for a behavioral response. But those theories often imply that the emotion feeling itself is a static state, neither growing nor waning in intensity, nor altering in character. If emotions were truly like this, it is not music but painting or sculpture—or photographs of faces—that would be the language of emotion.

Following a series of pleas for research on temporal sequences in affect (Frijda, 2007; Ebner-Priemer and Trull, 2009; Larsen et al., 2009), researchers have begun to analyze affective patterns over time (Kuppens, 2015). The major tools used in these studies are recollections collected at the end of a day or week, or real-time ambulatory assessments usually involving telephone prompts and responses. Typically, affect dynamic studies assess positive affect (PA) and negative affect (NA) repeatedly over the course of a day or week; data are summarized in terms of theoretically-based statistical indices, such as intrindividual variability; and the indices are used to predict psychological well-being (Houben et al., 2015) or psychiatric diagnoses (Santangelo et al., 2016).

Unfortunately, results to date from these studies have been meager. Dejonckheere et al. (2019) reanalyzed a series of studies and showed that “all affect dynamic measures add little to the prediction of psychological well-being once the explanatory power of mean levels of PA and NA is taken into account” (p. 483). They concluded that “conventional emotion research is currently unable to demonstrate independent relations between affect dynamics and psychological well-being” (p 478). It is understandable that contemporary emotion researchers would turn to statistical indices to quantify emotion dynamics, but at this stage of research it may be more useful to describe emotion patterns qualitatively, and for this music may offer some hints to a way forward. Some steps have been taken in studies of prosody, in Clynes’s (1977) notion of sentics, and in Frijda’s (2007) discussion of emotion episodes.

### Prosody

One area of emotion research that has explicitly drawn upon music is the study of emotional prosody (Quinto et al., 2013; Weniger et al., 2013). *Prosody* refers to the acoustic features of speech—pitch, loudness, rate, and so on—that are determined in part by semantics (as in the rising pitch at the end of a question) and in part by the emotion the utterance expresses (as in the harsh timbre and clipped words of a verbal threat). Prosody has been widely studied, having applications in fields ranging from speech pathology to human/robot interactions. Students of prosody have often used research methods that parallel those used in studies of music (e.g., Coutinho and Dibben, 2013). Results show that the acoustic cues that express a particular emotion in music generally express the same emotion in speech (Weniger et al., 2013). This phenomenon has long been recognized, and theorists from Spencer (1857) to Juslin (2013) have argued that musical expression mimics emotional prosody, which in turn reflects the underlying physiology of emotion: Slow music sounds sad because it resembles the slow speech caused by psychomotor retardation of depressed people.

### Sentics

Manfred Clynes was a concert pianist whose colleagues included Yehudi Menuhin and Pablo Casals; an engineer, inventor, and neologist who coined the term “cyborg;” and a neuropsychologist who co-edited a volume on emotions with Jaak Panksepp (Clynes and Panksepp, 1988). But his theory of *sentics*—his term for emotion—and the associated therapy, *sentic cycles* (Clynes, 1977), are far from mainstream. Only a handful of independent replications of his work have been reported, with mixed results (Nettelbeck et al., 1989; Hama and Tsuda, 1990). Some of his ideas seem relevant only to a rarified group of musicians or artists, and some of his claims strain credulity—practicing sentic cycles is said to cure anxiety, prevent suicide, and increase energy and creativity.

Nevertheless, his ideas merit consideration. Calling on great musicians as expert witnesses in the communication of emotion seems like a useful strategy, at least for the generation of hypotheses. Clynes’s (1992) focus on the temporal shape of emotions is surely appropriate. And his basic findings have a certain plausibility.

Sentics, from the Latin *sentire*, to feel, is Clynes’s term for emotion. He argued that the experience of emotion is inevitably tied to its expression or perception, so the natural unit of emotion has the duration of a single expression—a smile, a shout, a sigh. Longer emotion episodes are compounded of repeated instances. Clynes focused on basic emotions, and argued that each has a distinctive temporal form, regardless of the modality by which it is expressed (tone of voice, facial expression, musical phrase).

His procedure for detecting that form employs a sentograph, a pressure gauge on which participants rest their middle finger; the finger remains on the finger rest throughout the procedure. The pressure gauge is wired to a computer that records data and produces graphs of pressure-by-time curves. Participants are instructed to express a given emotion on a signal by pressing their finger once. (Recall that Clynes was a pianist, for whom the pressure of fingers is virtually the only way to express musical emotion). Signals are given by the experimenter at random intervals of a few seconds, and pressure is recorded both vertically (up vs. down) and horizontally (i.e., inward vs. outward). Each response is called an E-acton. When averaged over 50 trials, characteristic vertical and horizontal curves are obtained, distinct for each emotion, but generalizable across persons and cultures (at least in Clynes’s own research).

Consider three examples. Over a period of 2 s, an individual expressing anger gives a quick, sharp press down and outward that is quickly released. It is hardly a stretch to call this a jab. In contrast, an individual expressing love gives a slow, gentle touch that fades away; there is a slight motion inward. Joy is seen in a quick downward press that rebounds upward—a jump for joy. These are intuitively reasonable results, comparable to the findings that anger is expressed musically by loud and staccato sounds, and sadness by low and slow melodies (Gabrielsson and Lindström, 2001). What Clynes added was a specific temporal sequence and duration.

Many students of prosody have datasets in which the acoustic properties of utterances are recorded over time, and the duration of a typical sentence or pseudo-utterance is comparable to the duration of an E-acton. Clynes’ curves thus provide specific hypotheses that might be tested with available data. Do the pitch, or loudness, or rate contours of emotional sentences match his predictions?

In a sentic cycle, an individual seated at a sentograph is prompted to express (with finger pressure) a series of emotions in a fixed order: anger, hate, grief, love, sexual desire, joy, and reverence (Clynes, 1977). Multiple trials are given for each emotion before moving to the next. About 4 min is allotted to each emotion, so a single session requires about one-half hour. After 4 min, the felt emotion begins to fade—a phenomenon Clynes (1988) attributed to saturation of its neurohormonal substrate. Participants find it easy to switch to a new emotion, and any emotion can succeed any other, but with some carry-over effects: “Each state appears to cast its shadow on the following ones” (Clynes, 1977, p. 147).

### Episodes, Themes, and Threads

In his chapter on “Time,” Frijda (2007) made a great deal of a dissertation by Sonnemans (1991), because it was a rare and early source of information on the duration of emotions. Sonnemans asked informants to recall an emotion they had had the previous week and to describe what caused it, how they felt, and how the experience unfolded over time; informants created a graph of feeling intensity over time. Many of these accounts covered hours or days; many included more than one feeling intensity peak; and many mentioned a number of different emotions, simultaneously or successively, as part of the experience. For example, a woman reported on her response to a demeaning catcall: Over the course of 11 h she felt disgusted, angry, and humiliated and had disturbing dreams; the next morning, however, she thought the harasser had shown himself ridiculous and laughed the incident off.

Frijda (2007) called such experiences *emotion episodes*, which is perhaps an unfortunate word choice. Other researchers (e.g., Konečni, 2008) use the term “episode” quite differently, to refer to a single occurrence of a basic emotion, many of which might be included in each of the experiences Sonnemans (1991) analyzed. In literature, an “episode” is typically one of a series of distinct but coordinate units, such as chapters—but Frijda’s emotion episodes presumably are often isolated incidents, not necessarily continuous in time.

For musicians, the lexical ambiguity is even more awkward: A musical *episode* in a fugue is an incidental diversion from the ongoing development of the theme, whereas Frijda’s concern is precisely with the developing emotional experience. From a musical perspective, what Frijda described is in fact the analog of a *theme*, a recognizable tune that appears and disappears over the course of development, that (particularly in Romantic music) is emotionally expressive, and that often evolves, changing from soft to loud or from major to minor in successive statements. Frijda’s phenomenon is a series of emotions bound together by a common theme (e.g., reaction to a catcall).

However, the word *theme* is at least as ambiguous as *episode*, so I suggest this phenomenon be called an emotion *thread*. A plot thread is the narrative parallel to a musical theme: a distinct subplot that can appear, disappear, and reappear; evolve in tone or significance; and interact with other plot threads. When people are asked to recall emotional experiences, Sonnemans’s (1991) findings suggest that they organize them by emotion threads. If so, this poses yet another challenge to ambulatory assessment: Perhaps researchers need to sample feelings when, and only when, a particular thread is activated—which may be difficult to predict.

## Temporal Forms in Music and Affect

Music analysts have a great deal to say about the temporal structure of music, from chord progressions to the sequence of movements in a symphony. Imberty (2011) discussed parallels between music and narrative, noting that musical works have plots, with a beginning, complications, and resolutions; the tensions generated by this sequence compel the listener’s attention. Meyer (1956, 2001) argued that the central feature of music is a series of expectations, which are either gratifyingly fulfilled or cleverly disrupted. Narmour (1991) elaborated this idea, noting that the expectations may be bottom-up—based on universal principles of perception identified by Gestalt psychology—or top-down, based on learned musical conventions. The resulting musical narratives can and usually do express emotions. Zbikowski (2010) argued that music is ideally suited to that task because of “the resources it offers for simulating the progress of emotional states, and the ways it is able to represent rapid changes between such states” (p. 38).

If, as Langer (1957) argued, music is better suited to the depiction of emotions than words are, the psychology of affect can best be presented, not in a scientific theory, but in a musical composition. This is, of course, routinely done by composers who set words to music or write scores for films.

Musicologists distinguish between strophic and through-composed (*durchkomponiert*) music. The former uses a single melody for several verses, and so cannot be tailored to the nuances of each different verse. In such cases it seems reasonable to say that the music expresses the prevailing mood of the piece: “Yankee Doodle” is a jaunty tune; “Amazing Grace” is serene. Through-composed music, found chiefly in lieder and opera (notably Debussy’s *Pelleas et Melisande*), tries to shape music to the unfolding story, and so directly parallels the evolution of emotions.

Cohen (2001) described how film scores intensify and clarify the emotional content of a film. Imagine an application of this process to a psychological analysis. What would be learned if a composer were asked to provide a score for the videotape of a psychotherapy session? Both patient and therapist might gain a better understanding of how the patient deals with emotional issues. But to qualify as conventional psychology, this stream of affect would need to be translated into words, which would require a vocabulary for describing emotional sequences. I will discuss three concepts that may be useful for that purpose: affect contours, volitional affects, and affect transitions.

All three concepts would be classified by Juslin (2013) as *intrinsic*, involving “internal syntactic relationships within the music itself” that use changing levels of tension and release to express complex emotions such as hope and relief. As he noted, “(i)ntrinsic sources of musical expression have rarely been investigated thus far” (p. 9), in part because they would generally require the use of longer musical passages than are typically studied.

### Affect Contours

As long ago as 1929, Köhler proposed that psychologists adopt musical terminology: “the inner processes, whether emotional or intellectual, show types of development which may be given names such as *crescendo* and *diminuendo, accelerando* and *ritardando*” (Köhler, 1929, pp. 248–249, quoted in Langer, 1957). Such developments correspond to what Mayer et al. (2000) called *progressions* in emotion and to Kuppens and Verduyn (2017) *intensity profile shape*. Köhler’s notion has been extended by Stern (1985) under the name “vitality affects” or “vitality contours” (Stern, 1999).

Stern pointed out that emotions have a temporal shape that can be described by such terms as *surging, fading away, fleeting*, and *explosive*. He considered vitality affects to be entirely distinct from basic emotions, capturing the manner in which experience was felt rather than the content: Joy, anger, fear, or excitement may all fade away.

As Køppe et al. (2008) complained, in the course of his writings Stern used a variety of terms for vitality affects and extended their scope beyond affect to include temporal patterns of behavior, cognition, and motivation. In his last work on the topic, Stern (2010) called them “dynamic forms of vitality,” and defined them as a Gestalt of “movement, time, force, space, and intention/directionality” (p. 4) that gives the feeling of being alive. For the purposes of this article, a more limited definition is useful. I will use *affect contours* to refer to “the continual shifts in arousal, activation, and hedonics occurring split-second-by-split-second” (Stern, 1999, p. 70). Note that these are actually not new affects, but descriptions of the temporal course of the familiar dimensions of affect.

Stern, a developmental psychoanalyst who was chiefly concerned with the details of interactions between infant and parent, believed that these experiences have a very brief duration, “rarely over 5 s” (Stern, 1999, p. 68). If so, an extended expression of emotion, such as a full movement from a symphony might show, would need to be construed as a chain of affect contours.

**FIGURE 1:**
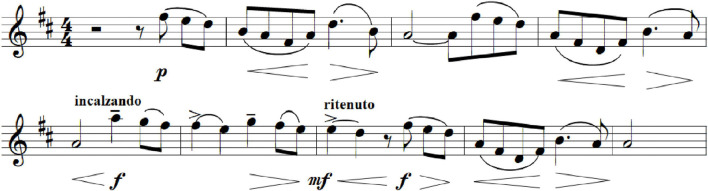
Andante from the 1st movement, Tchaikovsky’s *Pathétique* Symphony, measures 89–97 (Violin I). Audio version, Musopen Orchestra, Supplementary Audio 1.

Consider a musical example. The emotional tone of the Andante “*teneramente, molto cantabile, con espressione*” in Tchaikovsky’s *Pathétique* Symphony (Figure 1) is shaped by many musical features: the pure diatonic melody over a chromatic harmony, the major mode, the timbres of horns and muted strings. But the passage is brought to life—given vitality—by musical versions of affect contours. Beginning *piano*, the volume swells and sinks gently in the second measure and again in the fourth, creating a wistful, sighing effect. The fifth measure (*incalzando*, i.e., urgent, pressing) shows a rapid increase in volume and tempo, a surge of passion and longing that dissipates, at least in tempo, in the seventh measure. The seventh and eighth measures repeat the melody of the third and fourth, but now, played *forte*, they have a noble strength and dignity.

Although both Clynes and Stern were concerned with temporal forms over a brief interval, they differ fundamentally on the fixity of form. For Clynes, anger is always expressed by the equivalent of a jab, although the intensity of the jab may vary; for Stern, anger might be smoldering or explosive or undulating. Affect contours do not define basic affects, they merely shape them.

### Volitional Affects

The flow of music is structured by expectations and outcomes (Meyer, 1956, 2001; Narmour, 1991; Huron, 2008), which Sloboda and Juslin (2001) call *intrinsic* sources of emotion. Perhaps the most basic of these (at least to Western ears) is the chord progression from consonance to dissonance and back to consonance (e.g., I—V^7^—I), but any musical component (rhythm, melodic interval, harmonic density) that generates an expectation of some resolution creates (and can express) tension. The time scale may be quite extensive: The entire development section of a classical symphony creates a tension that is resolved by the return of the original theme and key in the recapitulation at the end of the movement.

**FIGURE 2:**
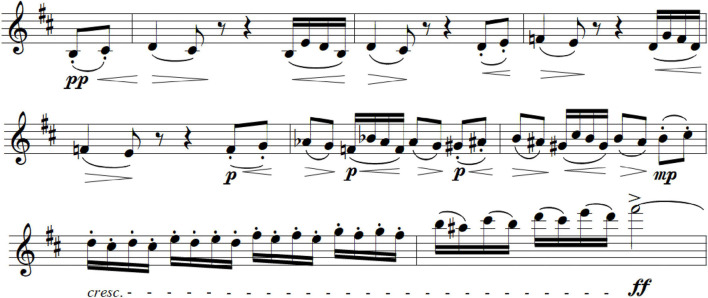
Allegro non-troppo from the 1st movement, Tchaikovsky’s *Pathétique* Symphony, measures 30–38 (Violin I). Audio version, Musopen Orchestra, Supplementary Audio 2.

A number of musical devices can be used to generate suspense. Figure 2 shows a passage from the Allegro non-troppo of the *Pathétique*’s first movement. Here a small motif is repeated in successively higher pitches; key modulations introduce uncertainty; phrasing is progressively compressed; and the volume increases dramatically in the last two measures before attaining the climatic F-sharp and a subsequent reduction in tension. The passage might be described as showing an increase in arousal—which it does—but there is also a sense of urgent seeking that is more than mere arousal.

The psychological basis of Meyer’s (1956) and Narmour’s (2000) theories of musical expectation is essentially perceptual-cognitive; a computer program could specify the musical expectations at any given moment in a musical passage. But from an affective perspective, these expectations can be understood as volitions: Listeners not only expect a dominant seventh chord to be resolved into the tonic; they *want* it to be resolved. It is this tension—the volitional pushes and pulls built into a composition—that holds the listener’s attention. The affective equivalents of musical expectation are expressed in such words as *longing, anticipation, suspense*, and *dread*, which might be termed volitional affects. Cowen et al. (2019, Figure 2) showed a cluster of emotions including *yearning*, *eagerness*, and *interest* that can be interpreted as positive volitional affects, and another group characterized by *detachment*, *boredom*, and *fatigue* that might be seen as negative volitional affects. (It is likely that negative volitional affects play a much larger role in human life than in music, because they would appear to have limited esthetic appeal).

It is rather puzzling that *suspense* has not been recognized as a basic emotion, like fear and anger; surely it is a common and powerful feeling. Under the guise of *tension*, it has occasionally appeared in dimensional models. Wundt (1902) included tension/relaxation (*Spannung/Lösung*) along with dimensions resembling valence and arousal, but most subsequent models—notably Russell’s (1980) circumplex—have omitted it. Because much research on music and emotion has been informed by Russell’s model, a crucial element of musical expression seems to have been missed. Eerola and Vuoskoski (2011) tried to correct this oversight by proposing that three dimensions (valence, energy arousal, and tension arousal) are needed to describe the emotion expressed in music, and one might suppose that tension arousal is related to the degree of expectation generated by the music. There is, however, a possible confusion here, because *tension* can mean either suspense (which can be resolved) or harsh unpleasantness (which can only be endured or ended). Eerola and Vuoskoski asked participants to rate excerpts from film scores and found that ratings of tension were strongly negatively correlated with valence (*r* = –0.83), suggesting that *tension* was interpreted by the raters to mean music that was ugly and jarring, suitable perhaps for horror films—indeed, a selection from the score of *Hellraiser* was one of those chosen to represent high tension. Compositions certainly do vary in the amount of musical suspense they generate, but this is presumably unrelated to valence; it is therefore essential to distinguish tension-as-distress from tension-as-suspense.

Suspense has been treated by appraisal theorists as a combination of hope and fear aroused by cognitive uncertainty (Ortony et al., 1988), and Madrigal and Bee (2005) provided evidence that both hope and fear occur and fluctuate when viewing a television advertisement deemed to be suspenseful. This would seem to suggest that suspense can be decomposed into the feeling dimensions of valence (hope vs. fear) and arousal (strong vs. weak) and the cognitive appraisal of outcome uncertainty. But in music, and presumably in life, there is also an affective component of suspense—something that feels like holding one’s breath—and it can be felt without either hope or fear. We sometimes want to know how something will turn out even when it is of no consequence to us.

Goal-directed striving is central to Carver and Scheier (1998) and Carver’s (2015) account of emotion, but the role of volition in that model is quite different from what is suggested here. Carver proposed that people monitor their progress toward goals (or away from adversity), and that the rate of progress is signaled by emotion: “Feelings with a positive valence mean that you are doing better at something than you need to, and feelings with a negative valence mean you are doing worse” (Carver, 2015, p. 302). Suspense is completely absent from this model, because it is precisely the feeling that accompanies uncertainty about whether (or when, or in what degree) you are doing better or worse.

The premise of this article—that music can offer a guide to understanding affect—is perfectly illustrated by the work of Huron (2008), who stated that “music provided me with a serendipitous starting place for theorizing more generally about the psychology of expectation” (p. viii). Huron was concerned with cognitive and physiological concomitants of expectation, but also centrally with emotion. He proposed a model in which five responses—imagination, tension, prediction, reaction, and appraisal (ITPRA)—account for expectation effects. *Imagination* consists of envisioning the consequences of a behavior and foretasting the pain or pleasure it would likely lead to; *tension* is an anticipatory arousal that is felt as suspense. After the event occurs, people feel a degree of satisfaction if their *prediction* was correct; this corresponds to the esthetic satisfaction that Meyer (1956) described when listeners correctly anticipate a musical event. The immediate, reflexive evaluation of an outcome Huron called the *reaction* response; its counterpart is the pleasant surprise that sabotaged musical expectations provide. The final affective state results from a conscious, reflective assessment of the event; this is the *appraisal* response, corresponding perhaps to the perceived overall affective tone of a piece of music. Huron’s (2008) book provides a detailed exposition of his model, with extensive musical examples.

### Affect Transitions

Music of a certain degree of complexity does not resolve all expectations in the obvious way. Instead, composers maintain the listener’s interest by changing the musical direction (Narmour, 1991) through key modulations, thematic variations, change in mode, and so on. Musical transitions, whether rapid or gradual, must be meaningful. For example, modulation—moving from one key (or tonality) to another—usually requires an intervening series of chords, each of which is compatible with the preceding and following chord, thus forming a chain that links the two keys. Clearly, such musical evolutions are akin to, and might express, what Mayer et al. (2000) called emotional transitions. If I have correctly interpreted the passage in Figure 1, it shows a transition from wistfulness through self-assertion to dignity.

The tempestuous Allegro non-troppo of the *Pathétique’s* first movement segues into the Andante shown in Figure 1 through sixteen bars of progressive slowing, quieting, and thinning of the orchestral texture (Musopen Orchestra, Supplementary Audio 3)—a transition based on affect contours. After the second statement of the Andante, the theme dies away to a passage marked *pppppp* (impossibly soft) for a solo bassoon, followed immediately by a fortissimo chord in the full orchestra (leading back to the “*feroce*” Allegro non-troppo). This too is a legitimate transition, in part because the soft and loud chords (D-major and C-minor sixth, respectively) form a meaningful, if unusual, harmonic progression, but it is an abrupt change that presents a dramatic surprise to the listener.

*Surprise* was considered by Ekman and Friesen (1971) to be a basic emotion. But Watson (2000) noted that surprise differs from other basic emotions in that it does not appear to correspond to a trait affect. People can be anger-prone or laughter-prone, but it appears that no one is surprise-prone. If asked, people can rate how often they experience surprise, but these ratings show little temporal stability, cross-observer agreement, or correlation with basic personality factors (Watson, 2000). Surprise appears to be not a basic emotion rooted in characteristics of the individual, but an affect transition inherent in the emotional experience itself (cf., Ortony, 2021).

Transitional affects are closely tied to emotion threads. The surprise fortissimo pivots from the Andante theme to the Allegro non-troppo theme—a transition across emotion threads. Figure 1 shows affect transitions within a theme: It is the continuity of the thread that allows us to perceive a set of different feelings (e.g., wistfulness, self-assertion, dignity) as different stages of the same emotional experience.

Appraisal theorists are likely to attribute affect transitions to reappraisals. In Frijda (2007) example of a woman whose feelings in response to a catcall were transformed from anger to amusement, the affective change was paralleled by a cognitive reappraisal: The harasser, who had first been seen as malicious, was reappraised as merely ridiculous. But the causal direction is not obvious here. It is entirely possible that the feelings of anger and disgust exhausted themselves in the course of a few hours through processes of affective adaptation, and the new attribution was merely a rationalization for the changed feelings. Indeed, the unmotivated affect expressed in music is problematic for appraisal theorists: Ellsworth and Scherer (2003) admitted that “neither appraisal theory nor any other current emotion theory can easily accommodate emotional responses to music” (p. 588).

## Some Directions for the Study of Affect in Time

Traditional studies of emotion—which ignore its temporal course—address a wide range of issues: Are emotions evolved and innate, or culturally acquired? When do they emerge developmentally? What are the physiological concomitants of emotion? the cognitive and interpersonal consequences? All these questions might be asked with regard to affect contours, volitional affects, and affect transitions. But surely we need to begin with description, to create a natural history of affect-through-time.

Pioneering researchers have offered a set of techniques for studying affective dynamics. Stern (2000) described a microanalytic interview in which a one-min section from a tape of a recently experienced event is reconstructed in detail by the interviewee. Schubert et al. (2012) assessed continuous ratings of emotion expressed by music by instructing respondents to move a computer mouse over a face representing the basic emotion they currently perceived. Carpenter et al. (2016) provided guidance in using experience sampling methods (or ambulatory assessment). Rachuri et al. (2010) developed an unobtrusive cell phone-based system that monitors tone of voice and infers emotional state. Here I will focus on the issues such methods might be used to investigate.

### Duration

Perhaps the most basic question is the duration of affects. Clynes (1977) and Stern (1999) argued that the natural unit of emotions is a few seconds, and Pöppel (1997) has argued that experience is integrated by the central nervous system into a subjective present of about 3 s. Emotion research on this micro time scale seems to be indicated. But emotion episodes are surely longer, and probably vary by type. Scherer and Wallbott (1994) found that fear, disgust, and shame tended to be short-lived, whereas joy and sadness could persist for hours or days. Watson (2000) studies of mood across hours and days suggests that, for most people, positive affect is a chronic state normally distributed around a personal mean, whereas negative affects are sporadic and thus show a skewed distribution.

For most affects, duration can be defined and assessed as the period from inception until “the intensity of the emotional response returns to zero or to a baseline level” (Verduyn et al., 2015, p. 331). But anticipation, dread, and suspense—volitional affects—potentially last from the recognition that an uncertain outcome is looming until it occurs, if ever. The outcome—a medical test result, a job offer—may be days or weeks away. Is the volitional affect continuous over that interval, perhaps backgrounded in consciousness, or does it recur when prompted by relevant cues, or surface spontaneously like intrusive post-traumatic thoughts?

### Vicissitudes and Metamorphoses

What is the typical course of an emotional experience? Emotions may grow in intensity, as when one works oneself up from annoyance to rage. Some affects—or their foregrounding in consciousness—may wax and wane periodically. Others may have an acute phase—say, the terror that a near-miss accident generates—and recur later as echoed apprehension. These patterns might be called the vicissitudes of affect.

But affects may also change qualitatively, morphing into new feelings. Reisch et al. (2008) used quarter-hourly assessment of borderline personality disorder patients to examine the sequence of specific emotions; they noted that compared to controls, the patients more frequently “switched from anxiety to sadness, from anxiety to anger, and from sadness to anxiety” (p. 42). Volitional affects are presumably resolved by the dreaded or desired outcome and succeeded by the appropriate basic emotion—despair or joy. Do positive and negative anticipations (hopes and fears) alternate, or does one predominate (see Madrigal and Bee, 2005)? Mayer et al. (2000) considered natural affective sequences as emotional transitions, and identified enough examples to construct a scale—though more extensive and systematic research on the nature and frequency of such spontaneous transitions is needed.

### Ambivalence and Equifinality

Emotional transitions in real life are complicated and ambivalent, because experienced affect is not monophonic, a simple sequence of successive feelings like notes in a melody. It is instead polyphonic, or homophonic, or bitonal, or cacophonic—that is, it is a continuously varying mixture of different feelings that may reinforce, contextualize, or clash with each other (cf., Larsen and Stastny, 2011). The trajectory of any single mood in this ongoing mix is surely affected by the other felt moods, which themselves may vary in their usual intrinsic duration. If Clynes (1977) was right, and “each state appears to cast its shadow on the following ones,” then the intricacies of affective patterns will be baffling.

Hollenstein (2015), however, has proposed that human affect is a complex system characterized by *attractors*. These are particular configurations of affects that tend to endure or recur regardless of the initial starting point—a phenomenon known as equifinality. The identification of such attractors—ideally in an affective space far more differentiated than the usual two-dimensional model—ought to be an aspiration of students of emotion.

### Individual Differences

One set of causes for affective attractors has already been identified: personality traits. Personality traits have long been linked to the frequency and intensity of affects (e.g., Costa and McCrae, 1980; Watson and Clark, 1992). There is some evidence that the durations of positive and negative affects (like their frequency and intensity) are linked to Extraversion and Neuroticism, respectively (Verduyn et al., 2015). Penner et al. (1994) found that there were consistent individual differences in the degree of variability of 11 affects, and these variances were themselves intercorrelated. Some people thus show greater emotional lability then others, and in extreme cases this may contribute to Borderline Personality Disorder (Ebner-Priemer et al., 2015).

## Some Considerations for the Study of Evolution

### Cultural Evolution

The fact that music seems designed for the expression of emotion does not imply that every musical utterance or style will be a perfect embodiment of emotion feelings. Humans have painted since they decorated Lascaux, but they did not master linear perspective—an accurate representation of the visual field—until the Renaissance. In the same way, there has been progress in the accurate musical expression of emotion, from simple keening to the intricate, extended, and nuanced portrayals of human passion in works like Wagner’s *Tristan and Isolde*.

Crocker (1966) traced the development of Western music from monophonic chant to the polyphonic motets and masses of the late Middle Ages. A particularly notable innovation of the 15th century was the shift from the open and neutral harmonies of fourths, fifths, and octaves to the emphasis on third and sixths, in both major (happy) and minor (unhappy) forms. Here at last was a musical structure that reflected the basic dimension of valence. Indeed, as Eerola et al. (2013) have shown, major or minor mode is the most important cue in the identification of the emotion expressed by a musical passage. If and when we have a full understanding of human affect, we will better be able to grasp the historical development of musical styles.

### Comparative Studies of Affect Dynamics

The evolution of music, like that of language, is a relatively recent phenomenon. The evolution of emotion is surely far more ancient, and one might surmise that the lessons offered here are of little relevance to an understanding of how affective systems arose. But the central argument advanced is that emotion is a temporal phenomenon, and its dynamics can and should be studied through cross-species comparisons.

Emotional reactions have durations in all species. The piloerection of a startled cat fades away, but how rapidly, with what contour, and with what adaptive significance? A dog awaiting the return of its owner surely feels the volitional effect of anticipation. Elated by the owner’s appearance, it eventually calms down; is this, as Clynes (1988) supposed, because of neurohormonal saturation? Certainly there are affect transitions in non-human animals: quarrels break out in social groups and are somehow resolved; infant distress is soothed by a parent; apathy turns to agitation when a threat appears. Are there commonalities in these processes across species, and if so, how far back in the evolutionary chain are they shared? A consideration of temporal sequences adds a new set of variables to the comparative study of emotion.

## Author Contributions

The author confirms being the sole contributor of this work and has approved it for publication.

## Conflict of Interest

The author declares that the research was conducted in the absence of any commercial or financial relationships that could be construed as a potential conflict of interest.

## Publisher’s Note

All claims expressed in this article are solely those of the authors and do not necessarily represent those of their affiliated organizations, or those of the publisher, the editors and the reviewers. Any product that may be evaluated in this article, or claim that may be made by its manufacturer, is not guaranteed or endorsed by the publisher.

## Supplementary Material

Supplementary Audio 1 Supplementary Material for “Music Lessons for the Study of Affect”: Figure 1. Andante from the 1st movement, Tchaikovsky’s *Pathétique* Symphony, measures 89-97. Audio version, Musopen Orchestra.

Supplementary Audio 2 Supplementary Material for “Music Lessons for the Study of Affect”: Figure 2. Allegro non-troppo from the 1st movement, Tchaikovsky’s *Pathétique* Symphony, measures 30-38. Audio version, Musopen Orchestra.

Supplementary Audio 3 Supplementary Material for “Music Lessons for the Study of Affect”: Allegro non-troppo from the 1st movement, Tchaikovsky’s *Pathétique* Symphony, measures 73-88. Audio version, Musopen Orchestra.

## References

[B1] Balkwill L. L. Thompson W. F. (1999). A cross-cultural investigation of the perception of emotion in music: Psychophysiological and cultural cues. *Mus. Percept*. 17 43–64.

[B2] Barrett L. F. Mesquita B. Ochsner K. N. Gross J. J. (2007). The experience of emotion. *Annual Rev. Psychol*. 58 373–403. 17002554 10.1146/annurev.psych.58.110405.085709 PMC1934613

[B3] Blood A. J. Zatorre R. (2001). Intensely pleasurable responses to music correlate with activity in brain regions implicated in reward and emotion. *Proc. Nat. Acad. Sci*. 98 11818–11823. 10.1073/pnas.191355898 11573015 PMC58814

[B4] Carpenter R. W. Wycoff A. M. Trull T. J. (2016). Ambulatory assessment: New adventures in characterizing dynamic processes. *Assessment* 23 414–424. 26887808 10.1177/1073191116632341 PMC6410721

[B5] Carver C. S. (2015). Control processes, priority management, and affective dynamics. *Emot. Rev*. 7 301–307.

[B6] Carver C. S. Scheier M. F. (1998). *On the Self-Regulation of Behavior.* New York, NY: Cambridge University Press.

[B7] Clynes M. (1977). *Sentics: The Touch of the Emotions.* Garden City, NY: Anchor Press.

[B8] Clynes M. (1988). “Generalised emotion, its production, and sentic cycle therapy,” in *Emotions and Psychopathology*, eds Clynes M. Panksepp J. (New York, NY: Plenum), 107–170. 10.1186/s13054-016-1208-6

[B9] Clynes M. (1992). “Time-forms, nature’s generators and communicators of emotion,” in *Reprinted from the Proceedings of the IEEE International Workshop on Robot and Human Communication*, (Tokyo).

[B10] Clynes M. Panksepp J. (eds) (1988). *Emotions and Psychopathology.* New York, NY: Plenum.

[B11] Cohen A. I. (2001). “Music as a source of emotion in film,” in *Music and Emotion: Theory and Research*, eds Juslin P. N. Sloboda J. A. (Oxford: Oxford University Press), 249–272.

[B12] Costa P. T. Jr. McCrae R. R. (1980). Influence of Extraversion and Neuroticism on subjective well-being: Happy and unhappy people. *J. Person. Soc. Psychol.* 38 668–678. 10.1037/0022-3514.38.4.668 7381680

[B13] Coutinho E. Dibben N. (2013). Psychoacoustic cues to emotion in speech prosody and music. *Cogn. Emot.* 27 658–684. 10.1080/02699931.2012.732559 23057507

[B14] Cowen A. Sauter D. Tracy J. L. Keltner D. (2019). Mapping the passions: Toward a high-dimensional taxonomy of emotional experience and expression. *Psychol. Sci. Public Interest* 20 69–90. 10.1177/1529100619850176 31313637 PMC6675572

[B15] Crocker R. L. (1966). *A History of Musical Style.* New York, NY: McGraw-Hill.

[B16] Damasio A. Carvalho G. B. (2013). The nature of feelings: Evolutionary and neurobiological origins. *Nat. Rev. Neurosci*. 14 143–152. 10.1038/nrn3403 23329161

[B17] Darwin C. (1871). *The Descent of Man and Selection in Relation to Sex.* London: John Murray.

[B18] Dejonckheere E. Mestdagh M. Houben M. Rutten I. Kuppens P. Tuerlinckx F. (2019). Complex affect dynamics add limited information to the prediction of psychological well-being. *Nat. Human Behav*. 3 478–491. 10.1038/s41562-019-0555-0 30988484

[B19] Ebner-Priemer U. W. Houben M. Santangelo P. Kleindienst N. Tuerlinckx F. Kuppens P. (2015). Unraveling affective dysregulation in Borderline Personality Disorder: A theoretical model and empirical evidence. *J. Abnorm. Psychol*. 124 186–198. 10.1037/abn0000021 25603359

[B20] Ebner-Priemer U. W. Trull T. J. (2009). Ecological momentary assessment of mood disorders and mood dysregulation. *Psychol. Assess*. 21 463–475. 10.1037/a0017075 19947781

[B21] Eerola T. (2010). Analysing emotions in Schubert’s Erlkönig: A computational approach. *Music Analysis* 29 214–233.

[B22] Eerola T. Friberg A. Bresin R. (2013). Emotional expression in music: Contribution, linearity, and additivity of primary musical cues. *Front. Psychol*. 4:487. 10.3389/fpsyg.2013.00487 23908642 PMC3726864

[B23] Eerola T. Vuoskoski J. K. (2011). A comparison of the discrete and dimensional models of emotion in music. *Psychol. Music* 39 18–49.

[B24] Eerola T. Vuoskoski J. K. (2013). A review of music and emotion studies: Approaches, emotion models, and stimuli. *Music Percept*. 30 307–340.

[B25] Ekman P. Friesen W. V. (1971). Constants across cultures in the face and emotion. *J. Person. Soc. Psychol*. 17 124–129. 10.1037/h0030377 5542557

[B26] Ellsworth P. C. Scherer K. R. (2003). “Appraisal Processes in Emotion,” in *Series in Affective Science. Handbook of Affective Sciences*, eds Davidson R. J. Scherer K. R. Goldsmith H. H. (Oxford: Oxford University Press), 572–595.

[B27] Frijda N. H. (2007). *The Laws of Emotion.* Hillsdale, NJ: Erlbaum.

[B28] Gabrielsson A. Lindström E. (2001). “The influence of musical structure on emotional expression,” in *Music and Emotion: Theory and Research*, eds Juslin P. N. Sloboda J. A. (Oxford: Oxford University Press), 223–248.

[B29] Gerardi G. M. Gerken L. (1995). The development of affective responses to modality and melodic contour. *Mus. Percept*. 12 279–290.

[B30] Greenberg D. M. Kosinski M. Stillwell D. J. Monteiro B. L. Levitin D. J. Rentfrow P. J. (2016). The song is you: Preferences for musical attribute dimensions reflect personality. *Soc. Psychologic. Person. Sci*. 7 597–605.

[B31] Habibi A. Damasio A. (2014). Music, feelings, and the human brain. *Psychomusicol. Music Mind Brain* 24 92–102. 10.1037/pmu0000033

[B32] Hama H. Tsuda K. (1990). Finger-pressure waveforms measured on Clynes’ sentograph distinguish among emotions. *Percept. Motor Skills* 70 371–376. 10.2466/pms.1990.70.2.371 2342835

[B33] Harris L. J. (2019). Does music matter? A look at the issues and evidence. *Dev. Neuropsychol*. 44 104–145. 10.1080/87565641.2016.1274316 29411997

[B34] Hevner K. (1936). Experimental studies of the elements of expression in music. *Amer. J. Psychol*. 48 246–268. 10.2307/1415746

[B35] Hollenstein T. (2015). This time, it’s real: Affective flexibility, time scales, feedback loops, and the regulation of emotion. *Emot. Rev*. 7 308–315.

[B36] Houben M. Van Den Noortgate W. Kuppens P. (2015). The relation between short-term emotion dynamics and psychological well-being: A meta-analysis. *Psychologic. Bull*. 141 901–930. 10.1037/a0038822 25822133

[B37] Hunter P. G. Schellenberg E. G. (2010). “Music and emotion,” in *Music Perception*, eds Jones M. R. Fay R. R. Popper A. N. (New York, NY: Springer), 129–164.

[B38] Huron D. (2008). *Sweet Anticipation: Music and the Psychology of Expectation.* Cambridge, MA: MIT press.

[B39] Imberty M. (2011). “Music, linguistics, and cognition,” in *Music and the Mind: Essays in Honour of John Sloboda*, eds Deliège I. Davidson J. Sloboda J. A. (Oxford: Oxford University Press), 3–16. 10.1093/acprof:osobl/9780199581566.003.0001

[B40] Izard C. E. (2009). Emotion theory and research: Highlights, unanswered questions, and emerging issues. *Annual Rev. Psychol*. 60 1–25. 10.1146/annurev.psych.60.110707.163539 18729725 PMC2723854

[B41] Juslin P. N. (2013). What does music express? Basic emotions and beyond. *Front. Psychol*. 4:596. 10.3389/fpsyg.2013.00596 24046758 PMC3764399

[B42] Juslin P. N. (2019). *Musical Emotions Explained: Unlocking the Secrets of Musical Affect.* Oxford: Oxford University Press.

[B43] Juslin P. N. Sloboda J. A. (eds) (2001). *Music and Emotion: Theory and Research.* Oxford: Oxford University Press.

[B44] Koelsch S. (2013). “Emotion and music,” in *Cambridge Handbook of Human Affective Neuroscience*, eds Armony J. Vuilleumier P. (Cambridge, MA: Cambridge University Press), 286–303.

[B45] Koelsch S. (2014). Brain correlates of music-evoked emotions. *Nat. Rev. Neurosci*. 15 170–180. 10.1038/nrn3666 24552785

[B46] Köhler W. (1929). *Gestalt Psychology.* New York, NY: Liveright.

[B47] Konečni V. J. (2008). Does music induce emotion? A theoretical and methodological analysis. *Psychol. Aesthetics Creativity* 2 115–129. 10.1037/1931-3896.2.2.115

[B48] Køppe S. Harder S. Væver M. (2008). Vitality affects. *Internat. Forum Psychoanal*. 17 169–179.

[B49] Kuppens P. (2015). It’s about time: A Special Section on affect dynamics. *Emotion Rev*. 7 297–300. 10.1177/1754073915590947

[B50] Kuppens P. Verduyn P. (2017). Emotion dynamics. *Cur. Opin. Psychol*. 17 22–26. 10.1016/j.copsyc.2017.06.004 28950968

[B51] Langer S. K. (1957). *Philosophy in a New Key: A Study in the Symbolism of Reason, Rite, and Art*, 3rd Edn. Cambridge, MA: Harvard University Press.

[B52] Larsen J. T. Stastny B. J. (2011). It’s a bittersweet symphony: Simultaneously mixed emotional responses to music with conflicting cues. *Emot.* 11 1469–1473. 10.1037/a0024081 21707144

[B53] Larsen R. J. Augustine A. A. Prizmic Z. (2009). A process approach to emotion and personality: Using time as a facet of data. *Cogn. Emot.* 23 1407–1426. 10.1080/02699930902851302

[B54] Lazarus R. S. (1966). *Psychological Stress and the Coping Process.* New York, NY: McGraw-Hill.

[B55] Madrigal R. Bee C. (2005). Suspense as an experience of mixed emotions: Feelings of hope and fear while watching suspenseful commercials. *Adv. Consumer Res*. 32 561–567.

[B56] Masataka N. (2009). The origins of language and the evolution of music: A comparative perspective. *Physics Life Rev*. 6 11–22. 10.1016/j.plrev.2008.08.003 22537940

[B57] Mayer J. D. Caruso D. R. Salovey P. (2000). Emotional intelligence meets traditional standards for an intelligence. *Intelligence* 27 267–298. 10.1016/s0160-2896(99)00016-1

[B58] McCrae R. R. (2007). Aesthetic chills as a universal marker of Openness to Experience. *Motivat. Emot.* 31 5–11. 10.1007/s11031-007-9053-1

[B59] McDermott J. (2008). The evolution of music. *Nat.* 453 287–288. 10.1038/453287a 18480798

[B60] Meyer L. B. (1956). *Emotion and Meaning in Music.* Chicago: University of Chicago Press.

[B61] Meyer L. B. (2001). “Music and emotion: Distinctions and uncertainty,” in *Music and Emotion: Theory and Research*, eds Juslin P. N. Sloboda J. A. (Oxford: Oxford University Press), 441–360.

[B62] Moors A. Ellsworth P. C. Scherer K. R. Frijda N. H. (2013). Appraisal theories of emotion: State of the art and future development. *Emot. Rev*. 5 119–124. 10.11124/JBISRIR-2016-003188 27941518

[B63] Narmour E. (1991). The top-down and bottom-up systems of musical implication: Building on Meyer’s theory of emotional syntax. *Music Percept.: Interdiscip. J*. 9 1–26. 10.2307/40286156

[B64] Narmour E. (2000). Music expectation by cognitive rule-mapping. *Music Percept.: Interdiscip. J*. 17 329–398. 10.2307/40285821

[B65] Nettelbeck T. Henderson C. Willson R. (1989). Communicating emotion through sound: An evaluation of Clynes’ theory of sentics. *Austral. J. Psychol*. 41 25–36.

[B66] Ortony A. (2021). Are all “basic emotions” emotions? A problem for the (basic) emotions construct. *Perspect*. *Psychologic. Sci*. 2021:1745691620985415. 10.1177/1745691620985415 34264141

[B67] Ortony A. Clore G. L. Collins A. (1988). *The Cognitive Structure of Emotions.* New York, NY: Cambridge University Press.

[B68] Patel A. D. (2010). *Music, Language, and the Brain.* Oxford: Oxford University Press.

[B69] Penner L. A. Shiffman S. Paty J. A. Fritzsche B. A. (1994). Individual differences in intraperson variability in mood. *J. Person. Soc. Psychol*. 66 712–721. 10.1037//0022-3514.66.4.712 8189348

[B70] Perlovsky L. (2010). Musical emotions: Functions, origins, evolution. *Phys. Life Rev*. 7 2–27. 20374916 10.1016/j.plrev.2009.11.001

[B71] Pinker S. (1997). *How the Mind Works.* New York, NY: Norton. 10.1111/j.1749-6632.1999.tb08538.x 10415890

[B72] Pöppel E. (1997). A hierarchical model of temporal perception. *Trends Cog. Sci*. 1 56–61. 10.1016/s1364-6613(97)01008-5 21223864

[B73] Pratt C. C. (1952). *Music as the Language of Emotion.* Washington, DC: US Government Printing Office.

[B74] Quinto L. Thompson W. F. Keating F. L. (2013). Emotional communication in speech and music: The role of melodic and rhythmic contrasts. *Front. Psychol*. 24:184. 10.3389/fpsyg.2013.00184 23630507 PMC3633948

[B75] Rachuri K. K. Musolesi M. Mascolo C. Rentfrow P. J. Longworth C. Aucinas A. (2010). “EmotionSense: a mobile phones based adaptive platform for experimental social psychology research,” in *Proceedings of the 12th ACM international conference on Ubiquitous computing*, (Association for Computing Machinery), 281–290.

[B76] Reisch T. Ebner−Priemer U. W. Tschacher W. Bohus M. Linehan M. M. (2008). Sequences of emotions in patients with borderline personality disorder. *Acta Psychiatrica Scand.* 118 42–48. 10.1111/j.1600-0447.2008.01222.x 18582346

[B77] Reybrouck M. Eerola T. (2017). Music and its inductive power: A psychobiological and evolutionary approach to musical emotions. *Front. Psychol*. 494:494. 10.3389/fpsyg.2017.00494 28421015 PMC5378764

[B78] Rozin P. Rozin A. (2018). Advancing understanding of the aesthetics of temporal sequences by combining some principles and practices in music and cuisine with psychology. *Perspect. Psychologic. Sci*. 13 598–617. 10.1177/1745691618762339 30040907

[B79] Russell J. A. (1980). A circumplex model of affect. *J. Person. Soc. Psychol*. 39 1161–1178. 10.1037/h0077714

[B80] Sachs M. E. Damasio A. Habibi A. (2015). The pleasures of sad music: A systematic review. *Front. Hum. Neurosci.* 9:404. 10.3389/fnhum.2015.00404 26257625 PMC4513245

[B81] Santangelo P. S. Limberger M. F. Stiglmayr C. Houben M. Coosemans J. Verleysen G. (2016). Analyzing subcomponents of affective dysregulation in borderline personality disorder in comparison to other clinical groups using multiple e-diary datasets. *Borderline Person. Disord. Emot. Dysregulation* 3 1–13. 10.1186/s40479-016-0039-z 27386138 PMC4934004

[B82] Savage P. Loui P. Tarr B. Schachner A. Glowacki L. Mithen S. (2020). Music as a coevolved system for social bonding. *Beh. Brain Sci*. 2020 1–36. 10.1017/S0140525X20000333 32814608

[B83] Scherer K. R. (2004). Which emotions can be induced by music? What are the underlying mechanisms? And how can we measure them? *J. New Mus. Res*. 33 239–251. 10.1080/0929821042000317822

[B84] Scherer K. R. Wallbott H. G. (1994). Evidence for universality and cultural variation of differential emotion response patterning. *J. Person. Soc. Psychol*. 66 310–328. 10.1037//0022-3514.66.2.310 8195988

[B85] Sherer K. R. Zentner M. (2008). Music evoked emotions are different—often more aesthetic than utilitarian. *Beh. Brain Sci*. 5 595–596. 10.1017/s0140525x08005505

[B86] Schopenhauer A. (1969). *The World as Will and Representation.* New York, NY: Dover Publications.

[B87] Schubert E. Ferguson S. Farrar N. Taylor D. McPherson G. E. (2012). *The six emotion-face clock as a tool for continuously rating discrete emotional response to music, in International Symposium on Computer Music Modeling and Retrieval.* Berlin: Springer, 1–18.

[B88] Sloboda J. A. Juslin P. N. (2001). “Psychological perspectives on music and emotion,” in *Music and Emotion: Theory and Research*, eds Juslin P. N. Sloboda J. A. (Oxford: Oxford University Press), 71–104.

[B89] Snowden C. T. Zimmermann E. Altenmüller E. (2015). Music evolution and neuroscience. *Prog. Brain Res*. 217 17–34. 25725908 10.1016/bs.pbr.2014.11.019

[B90] Sonnemans J. (1991). *Structure and Determinants of Emotional Intensity.* Unpublished doctoral dissertation. Amsterdam: University of Amsterdam.

[B91] Spencer H. (1857). The origin and function of music. *Fraser’s Mag*. 56 396–408.

[B92] Stern D. N. (1985). *The Interpersonal World of the Infant.* New York, NY: Basic Books.

[B93] Stern D. N. (1999). “Vitality contours: The temporal contour of feelings as a basic unit for constructing the infant’s social experience,” in *Understanding Others in the First Months of Life*, ed. Rochat P. (Mahwah, NJ: Erlbaum), 67–80.

[B94] Stern D. N. (2000). “Introduction to the paperback edition,” in *The Interpersonal World of the Infant*, ed. Stern D. N. (New York, NY: Basic Books). 10.15265/IYS-2016-s041

[B95] Stern D. N. (2010). *Forms of Vitality: Exploring Dynamic Experience in Psychology, the Arts, Psychotherapy and Development.* Oxford: Oxford University Press.

[B96] Swaminathan S. Schellenberg E. G. (2015). Current emotion research in music psychology. *Emot. Rev.* 7 189–197. 10.1177/1754073914558282

[B97] Thayer J. F. (1986). Multiple indicators of affective responses to music. *Dissertation Abstracts Internat.* 47:12.

[B98] Verduyn P. Delaveau P. Rotgé J.-Y. van Mechelen I. (2015). Determinants of emotion duration and underlying psychological and neural mechanisms. *Emot. Rev*. 7 330–335. 10.1177/1754073915590618

[B99] Watson D. (2000). *Mood and Temperament.* New York, NY: Guilford.

[B100] Watson D. Clark L. A. (1992). On traits and temperament: General and specific factors of emotional experience and their relation to the Five−Factor Model. *J. Person.* 60 441–476. 10.1111/j.1467-6494.1992.tb00980.x 1635050

[B101] Weniger F. Eyben F. Schuller B. W. Mortillaro M. Scherer K. R. (2013). On the acoustics of emotion in audio: What speech, music, and sound have in common. *Front. Psychol*. 4:292. 10.3389/fpsyg.2013.00292 23750144 PMC3664314

[B102] Wundt W. (1902). *Grundzüge des Physiologischen Psychologie*, 5th Edn, Vol. 2. Leipzig: Wilhelm Engelmann.

[B103] Zangwill N. (2004). Against emotion: Hanslick was right about music. *Brit. J. Aesthetics* 44 29–43. 10.1093/bjaesthetics/44.1.29

[B104] Zbikowski L. M. (2010). Music, emotion, analysis. *Music Anal.* 29 37–60. 10.1111/j.1468-2249.2011.00330.x

[B105] Zentner M. R. Grandjean D. Scherer K. R. (2008). Emotions evoked by the sound of music: Characterization, classification, and measurement. *Emo*. 8 494–521. 10.1037/1528-3542.8.4.494 18729581

[B106] Zentner M. R. Meylan S. Scherer K. R. (2000). “Exploring ‘musical emotions’ across five genres,” in *Paper presented at the Sixth International Conference of the Society for Music Perception and Cognition*, (Keele).

